# Impact of a medico-pharmaceutical follow-up and an optimized communication between hospital and community on the readmission to the emergency department for an adverse drug event: URGEIM, study protocol for a randomized controlled trial

**DOI:** 10.1186/s13063-021-05501-4

**Published:** 2021-08-06

**Authors:** Cyril Breuker, Marie Faucanié, Marion Laureau, Damien Perier, Véronique Pinzani, Grégory Marin, Mustapha Sebbane, M. Villiet

**Affiliations:** 1 CHU Montpellier, Clinical Pharmacy Departement, Univ. Montpellier, 34 295 Montpellier Cedex 5, France; 2 PhyMedExp, Univ Montpellier, CNRS, INSERM, Montpellier, France; 3 CHU Montpellier, Clinical Reasearch and Epidemiology Unit (Departement Information Médicale), Univ. Montpellier, 34 295 Montpellier Cedex 5, France; 4 CHU Montpellier, Emergency Medicine Department, Univ. Montpellier, 34 295 Montpellier Cedex 5, France; 5 CHU Montpellier, Medical Pharmacology and Toxicology Department, Univ. Montpellier, 34 295 Montpellier Cedex 5, France

**Keywords:** Emergency department, Pharmacist, Adverse drug event

## Abstract

**Background:**

Adverse drug events (ADE) represent one of the main causes of admission to emergency department (ED). Their detection, documentation, and reporting are essential to avoid readmission.

We hypothesize that a pharmacist-initiated multidisciplinary transition of care program combining ED pharmacist contribution and medications’ data transfer between inpatient and outpatient caregivers will reduce emergency visits related to ADE

**Method/design:**

This is a prospective, open-label, randomized controlled trial. The primary aim of the study is 6-month ED readmission related to the same ADE. Three hundred forty-six adult patients with an ADE detected by a binomial pharmacist-physician will be recruited from the ED of an University Hospital and will be randomized in two groups: [1] experimental group (multidisciplinary transition of care program and medications’ data transfer between inpatient and outpatient caregivers) and [2] control group (usual care). Patients will be followed up over a period of 6 months. Endpoints will be carried out blindly of the randomization arm. The primary endpoint is the rate of patients who had at least one readmission in the ED for the same reason at 6 months (data collected during a phone call with the patient and the general practitioner). Trials registered NCT03725046.

**Discussion:**

The trial results will have implications for the role of the clinical pharmacist in an emergency department. If successful, the intervention could be considered for implementation across other hospitals.

**Trial registration:**

ClinicalTrials.govNCT03725046. Registered on 30 October 2018

## Background

The hospital discharge process introduces care coordination challenges. A lot of patients, suffering from acute or chronic diseases, are evaluated to emergency departments for medical evaluation and subsequently discharged. On the border of hospital and community areas, emergency department (ED) realize a lot of medical consultations with a representative risk of communication failure with primary care providers. So many times, informative data failed to be transmitted to the general practitioners and are generally never transmitted to the community pharmacist.

Adverse drug events (ADE) represent one of the main causes of unplanned hospital admissions (up to 25%) and hospital deaths [1, 2]. ADE are defined as any patient damage, related to medication management and resulting from appropriate care, unsuitable care, or a lack of care. They are responsible for unfavorable clinical evolution and represent a major economic and healthcare problem [3, 4]. ADE require specific medication management to prevent any ADE recurrence and hospitalization.

Clinical pharmacist can improve transition of care (TOC), first by detecting ADE responsible of emergency visit and medication involved, then by making specific alert of primary care physicians improving medication management.

Many studies indicate that errors in TOC are common included detection and management of ADE [5–8]. Several strategies must be developed for limiting ADE recurrence, healthcare utilization, unplanned readmission, hospitalization, and/or death of the patients. Strategy to improve communication between healthcare providers is fundamental. Furthermore, pharmacists’ interventions (medication history end reconciliations, direct communication between providers and pharmacists, telephone follow-up with patients after discharge…) can limit ADE occurrence [1, 9–11].

Since November 2011, a pharmacy team is effectively integrated in ED of our hospital to help ED team about medication of inpatients: medication histories, ADE detection, and ADE management. We hypothesize that a pharmacist-initiated multidisciplinary TOC program combining ED pharmacist contribution and medications’ data transfer between inpatient and outpatient caregivers will reduce emergency visits related to ADE.

## Methods/design

### Aims of the study

The primary aim of the study is 6-month ED readmission related to the same ADE.

The secondary objectives are to evaluate the impact of this program at 6 months after admission for an ADE on:

- All-cause ED readmission,
- ADE and all-cause hospitalization,
- ADE and all-cause mortality,
- ADE and all-cause healthcare utilization (primary care and specialist post discharge appointments)
- Medication modification related to the ADE

We will also evaluate in the “Intervention group”: the impact the program at 6 months on:

- Satisfaction of community pharmacists and physicians
- ED pharmaceutical workload required to implement this discharge program.

### Study design

**Fig. 1:**
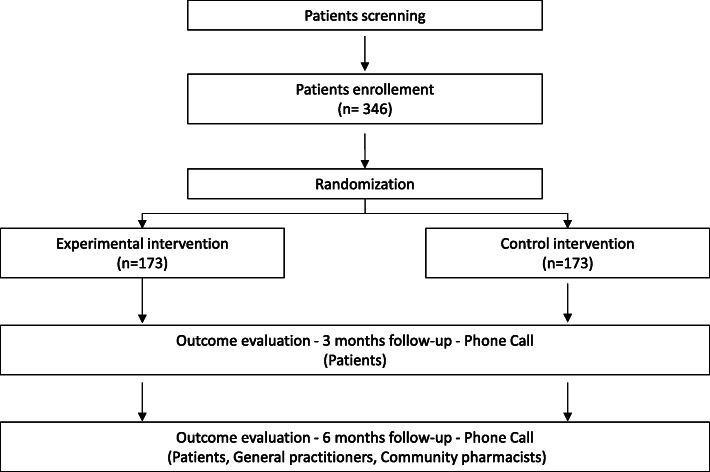
Study flow chart

This is a prospective, monocentric, open-label, parallel group, randomized controlled trial. Patients with ADE are recruited from the ED of the University Hospital of Montpellier, France. Eligible participants are randomized to TOC program or usual care group. There is a 6 months follow-up, and the evaluation of the study outcomes is carried out blindly of the randomization arm. The study flow chart is presented in Fig. 1.

### Eligibility criteria

The participants, identified through ED, are aged from 18 and have an ADE (1/adverse drug reaction (ADR) without misuse, 2/ADR related to misuse and 3/nonadherence with unfavorable clinical repercussions). Moreover, pharmaceutical consultation with the patient or their relatives must be achievable and informed consent must be signed prior to inclusion in the study. Non-inclusion criteria are as follows: inability to carry out the patient’s drug history, ADE linked to medical care in the ED or voluntary drug intoxication, and pregnancy. Drop outs during the study include consent withdrawal, continuation of the study prejudicial to the patient according to the investigator, loss to follow-up, death occurring before hospital discharge, and patient hospitalized during more than 2 months after the admission to the ED.

### Randomization

Participants meeting the inclusion criteria are randomized by the ED pharmacist using a centralized computer generated randomization available on the e-CRF. Minimization randomization is used. Randomization (1:1) is stratified on the following criteria: type of ADE (linked to medication vs noncompliance), severity of the ADE (discharge from ED vs hospitalization), and age (± 70 years old).

### Recruitment and intervention

#### Detection of ADE

In the ED of the University Hospital of Montpellier, pharmaceutical management is currently offered upon arrival. Thus, in addition to the usual medical consultation, a pharmaceutical consultation is done from 8:30 a.m. to 6 p.m., from Monday to Friday (standard of care in our center). During this pharmaceutical consultation, the pharmacist carries out the best possible medication history (BPMH). The BPMH was defined as the most comprehensive list of all medications taken by the patient, including prescription drugs and self-medications, and was based on at least three sources of information (patient or family interview; drug prescriptions, if available; phone calls to community pharmacies, general practitioner and/or nurses; medical record). The interview is conducted following the recommendations of the World Health Organization [12]. ADE are detected based on the French method of pharmacovigilance imputation [13].

ADE are defined as any patient damage, related to medication management and resulting from appropriate care, unsuitable care, or a lack of care [4]. This definition included injuries (signs, symptoms or laboratory abnormalities) resulting from ADR or nonadherence with bad issue. Voluntary medication poisoning was excluded from the ADE definition. Event’s severity was classified into two categories: ED discharge or hospitalization.

Before participating in the study, physicians and pharmacists received training in detecting ADE. This training is provided by a multidisciplinary expert committee including one pharmacovigilant physician, one emergency physician, and one pharmacist. This group of experts has been collaborating since 2011 on ADE detection. In case of doubt about the ADE detection, the expert committee can be consulted.

When ADE is detected, the ED pharmacist describes the study to the patient. Subjects willing to participate sign an informed consent form before the baseline evaluation, data collection and randomization.

#### Experimental intervention

**Fig. 2:**
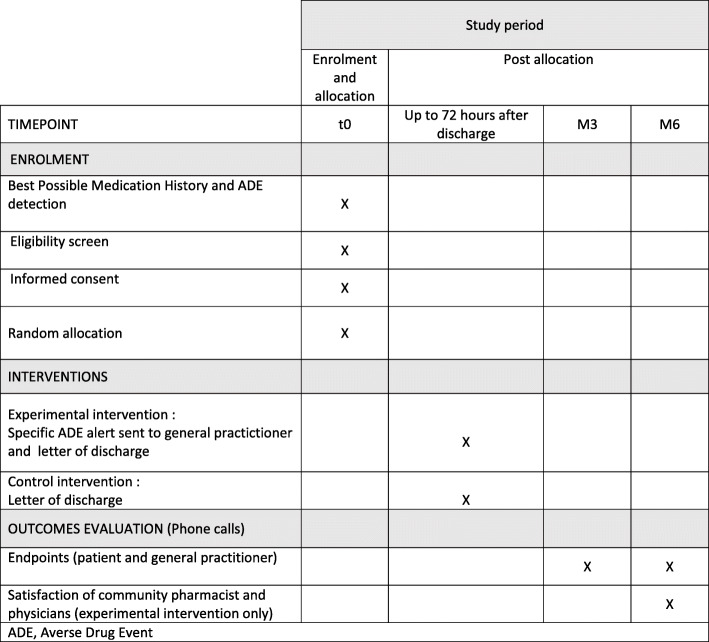
Schedule of enrolment, intervention, and assessments

ED pharmacist makes a 72-h post discharge alert using telephone call. This follow-up call improves direct communication between the general practitioner and the community pharmacist of the patient and is an opportunity to discuss ADE management. A specific ADE alert leading to ED visit is sent to the general practitioner in addition to the letter of discharge (usual care). The specific ADE alert is written and validated by the ED physician and ED pharmacist, including ADE type and suspected medication as well as any recommendation (therapeutic modification, referral to specialized consultations, names and contact information to easily reach the physician and the pharmacist involved in the treatment charge in the ED) (see Fig. 2).

#### Control intervention

A letter of discharge specifying the reason for emergency admission is sent to the general practitioner (standard of care).

### Outcomes

Outcomes will be collected during a phone call by a pharmacist blindly of the randomization arm, with the patient at 3 and 6 months and with general practitioner at 6 months. When available, outcomes will be confronted with data from the patient’s electronic medical file. The phone call at 3 months avoids the risk of loss of contact with patients.

The primary endpoint is the rate of patients with at least one same ADE leading to ED readmission at 6 months.

Secondary endpoints evaluated at 6 months are:

- Rate of patients who had at least one all-cause ED readmission
- Rate of patients who had at least one ADE hospitalization and all-cause hospitalization
- Rate of potentially preventable hospitalizations based on the definition of the American agency AHRQ adapted to French data [14]
- Rate of ADE and all-cause death
- Rate of ADE and all-cause medical consultations (general practitioner and specialists)
- Rate of medication modification.

In the intervention group, we will also collect the satisfaction of healthcare referrals (general practitioners and pharmacist) evaluated with a Likert-like scale and pharmaceutical time required to implement the intervention.

We will also collect patients’ characteristics (sociodemographic data, comorbidities), medications (name, dosage, compliance, modification during the last 15 days), characteristics of ADE (type, severity), and, for the interventional group, the type and number of pharmacist recommendations.

An electronic case report form (e-CRF) is developed by the Clinical Research and Epidemiology Unit from the software Ennov Clinical® to collect and to control data quality at entry and during the trial. Data collected in the e-CRF are pseudo-anonymized (subject identification code and subject initials).

### Harms

The investigator is responsible for collecting all the adverse events throughout the study. They will be assessed at each visit and reported in the e-CRF. Harms reporting will be carried out in compliance with the French regulations in force throughout the study. There are no anticipated harms. The sponsor of the study takes out insurance for the entire duration of the study guaranteeing its own civil liability as well as that of any party involved in the study.

### Monitoring and audit

On-site or remote monitoring visits will be realized by the monitors designated by the sponsor to check compliance with the protocol, the completeness, accuracy and consistency of the data, and adherence to Good Clinical Practice (GCP). During the trial, an audit could be organized by the sponsor or competent authority. The audit can be applied at all stages of research, from protocol development to publication of results. This study does not have a data monitoring committee due to low risk interventions.

### Sample size

The study by Zhang M et al. showed 17% of hospital ADE readmission. This rate is based on data from medical coding which probably underestimate this rate [15]. A descriptive pre-study carried out in the study center to assess the impact of specific ADE alert to community health professionals in case of ADE showed a rate of emergency readmissions of 13.7% without transmission of the medico-pharmaceutical report and a rate of 5.1% with transmission. Assuming a 14% readmission rate in the control group and a rate of 5% in the TOC group, a sample size of 314 subjects is required with two-sided alpha = 0.05 and a power of 80%. To take into account a 10% dropout rate, 173 subjects per group will be included.

### Statistics and data analysis

The statistical analysis will be performed by the Clinical Research and Epidemiology Unit at the Montpellier University Hospital using SAS software (SAS Institute, Cary, NC, USA). The level of significance is set at *p* < 0.05 (bilateral).

Statistical analysis will be performed on the intention to treat (ITT) population. The decision to perform analyses on other population sets for sensitivity analysis will be taken by the study methodologist if necessary.

The number of screened, included, and randomized patients in each group will be summarized in a flow chart. Excluded and lost to follow-up patients will be reported.

Patients’ characteristics in the total population and in each group will first be analyzed. Quantitative variables will be expressed by mean, standard deviation, median, range, and quartiles. Qualitative and categorical data will be summarized by frequencies and percentages. Characteristics will be compared between the two groups using Student’s *t* test or Mann-Whiney *U* test for continuous variables and chi-square or Fisher exact test for categorical variables.

The distribution of the main outcome will be compared between the two groups with chi-square or Fisher exact test. Logistic regression models will also be implemented to analyze the effect of the group on the main outcome by adjusting on variables of interest. Univariate models will first be executed, and the variables with a *p* value lower than 0.15 will be considered for a multivariable model. Then, the variables with a *p* value lower than 0.05 in the multivariable model after a stepwise selection of variables will be considered statistically significant.

Categorical secondary outcomes will be analyzed using the same methodology as the main outcome, with chi-square or Fisher exact test and logistic regression models. Quantitative secondary outcomes will be compared between the two groups using Mann-Whiney *U* test, along with multivariable linear regressions with covariates of interest.

### Missing data

The type of missing data will be determined for the main variables of interest. Conditional rejection of the hypothesis NMAR (not missing at random) and the assumption M.C.A.R. (missing completely at random) against MAR (missing at random) will be tested by studying the relationship between the variable status (missing or not) and the values of the covariates. If the missing data is type MAR or MCAR, a multiple imputation will be implemented [16]. When possible, missing data will be complemented by the deductive method from all of the variables collected. These multiple imputations will be used in the sensitivity analysis.

## Discussion

ADE definition differed among studies and sometimes overlapped with other terms, such as drug-related problems (including, subtherapeutic and supratherapeutic doses, untreated indication, drug without indication…), drug-related visit, or medication errors. An interprofessional variation in adverse drug definition can explain these findings [17, 18]. Physicians generally accept narrower definitions than pharmacist [5]. However, all the studies point out that regardless of the definition used, the main adverse drug events found are ADR and nonadherence [5]. That is why we choose de define the ADE as an effect resulting from a side-effect without misuse, a side effect with misuse and nonadherence with unfavorable clinical repercussions.

Pharmacist and emergency physicians of the University Hospital of Montpellier work together to detect ADE since 2011. A pharmaceutical team, supervised by the principal investigator of the study, is integrated in the ED. This team is responsible for conducting pharmaceutical consultations focused on patient’s medications, in connection with the medical team. The objectives of these consultations are to achieve an accurate medication history and to detect ADE. The implementation of the URGEIM study aims to show that the integration of a clinical pharmacist into a medical team in ED allows individual support for patients undergoing ADE and thus reduces risk of recurrence of ADE.

This multidisciplinary system including a clinical pharmacist will also permit:

- To sensitize general practitioner about drug iatrogenicity
- To improve the community/hospital link providing a rapid feedback of reliable information and recommendations to community healthcare providers.
- To sensitize and involve community pharmacists in monitoring patients with medication risks.

The originality of our study is [1] to optimize the ADE detection by pharmacist carrying out an exhaustive medication history and [2] to develop the community-hospital link to improve patient care presenting in ED with ADE.

The URGEIM trial builds on existing healthcare organizations. As such, this organization can be generalized in any center hospital wishing to reduce ADE.

## Trial status

The trial is currently in the recruitment phase. The first enrolment occurred on 19 November 2018. This the 3rd version of the protocol (version 3, 13 May 2020) approved by the ethics committee on 24 June 2020. At August 2020, 288 patients have been included in the study. We anticipate completing study by the end of 2021.

## Data Availability

Dataset will be made available under conditions to persons who address a reasonable request to the coordinating investigator. The results of the study will be presented to healthcare professionals during conferences and valorized through publications. Study participants will be informed of the results upon request.

## Abbreviations

ADE: Adverse drug event
ADR: Adverse drug reaction
BPMH: Best possible medication history
ED: Emergency department
ITT: Intention to treat
TOC: Transition of care
